# Gamma noise to non-invasively monitor nuclear research reactors

**DOI:** 10.1038/s41598-024-59127-y

**Published:** 2024-04-10

**Authors:** Oskari Pakari, Tom Mager, Pavel Frajtag, Andreas Pautz, Vincent Lamirand

**Affiliations:** 1 Laboratory for Reactor Physics and Systems Behaviour, Ecole polytechnique fédérale de Lausanne, 1015 Lausanne, Switzerland; 2 Nuclear Energy and Safety Division, Paul Scherrer Institut, 5232 Villigen PSI, Switzerland

**Keywords:** Nuclear physics, Techniques and instrumentation

## Abstract

Autonomous nuclear reactor monitoring is a key aspect of the International Atomic Energy Agency’s strategy to ensure nonproliferation treaty compliance. From the rise of small modular reactor technology, decentralized nuclear reactor fleets may strain the capacities of such monitoring and requires new approaches. We demonstrate the superior capabilities of a gamma detection system to monitor the criticality of a zero power nuclear reactor from beyond typical vessel boundaries, offering a powerful alternative to neutron-based systems by providing direct information on fission chain propagation. Using the case example of the research reactor CROCUS, we demonstrate how two bismuth germanate scintillators placed outside the reactor vessel can precisely observe reactor criticality using so called noise methods and provide core status information *in seconds*. Our results indicate a wide range of applications due to the newly gained geometric flexibility that could find use in fields beyond nuclear safety.

## Introduction

In dealing with climate crisis scenarios^1^, nuclear energy may play a key role in providing low-emission electricity to a world that seeks to weaken its dependency on fossil fuels^2^. Across the spectrum of plausible pathways until 2060, the World Energy Council’s World energy scenarios report of 2019^3^ shows throughout an increase in the absolute amount of installed nuclear power.

Nuclear energy faces nonetheless the dichotomy of being an inherently strategic technology, with special nuclear material (SNM) being potentially available from the fuel cycle. Efforts of nonproliferation as spearheaded by the International Atomic Energy Agency (IAEA) thus aim to ensure the peaceful use of nuclear energy, e.g. via controlled technology transfer to non-nuclear states, treaty facilitation and verification, as well as inspections and monitoring of nuclear facilities around the world. The safeguard mission under the nonproliferation treaty is specifically to detect the diversion of declared nuclear materials, the misuse of nuclear facilities, as well as undeclared nuclear activities^4^.

The projected growth in installed nuclear power therefore demands an increase in safeguards efforts. In the context of inspections and reactor monitoring, it is part of the IAEA’s R &D plans^5^ to improve current safeguard technologies to effectively follow recent technological trends. This includes in particular small modular reactors (SMR), which due to their smaller size and design require novel approaches for nonproliferation monitoring^6^. The measures to meet the safeguards mission often require an inspector to conduct item identification and counting to manually confirm the declared inventory of a facility. Autonomous monitoring of nuclear reactors using radiation signatures is therefore a key development avenue.

Radiation signatures from nuclear reactors that are commonly used for monitoring include neutrons, gamma rays, and antineutrinos. Large-area neutron detectors for stand-off distance (i.e. beyond the reactor vessel) have recently been shown to be able to follow a reactor’s power evolution and detect power diversions on the order of tens of minutes^7^. The information of the reactor’s exact state is however unknown, and the information may be tampered with by deliberate source positioning. Similarly, antineutrino detectors have been shown to be able to detect a reactor’s power evolution, with the added information of the antineutrino spectrum being dependent on the fuel composition^8^ to detect fuel exchanges that may indicate secondary use of the reactor. Measurements of antineutrino spectra are however still in infancy^9^ and subject to large uncertainties, and the speed of information is of the order of weeks and typically applicable only to power systems above >100 MW_th_. In addition, detector volumes of 3000 liters or more are required to reach significant detection rates^10^, pointing towards cost and mobility challenges. Both neutron and antineutrino detectors are therefore not able to measure fuel composition changes that occur on a faster than week-long scale, leaving time for second use activities and signal tampering in smaller and low-power facilities.

**Figure 1:**
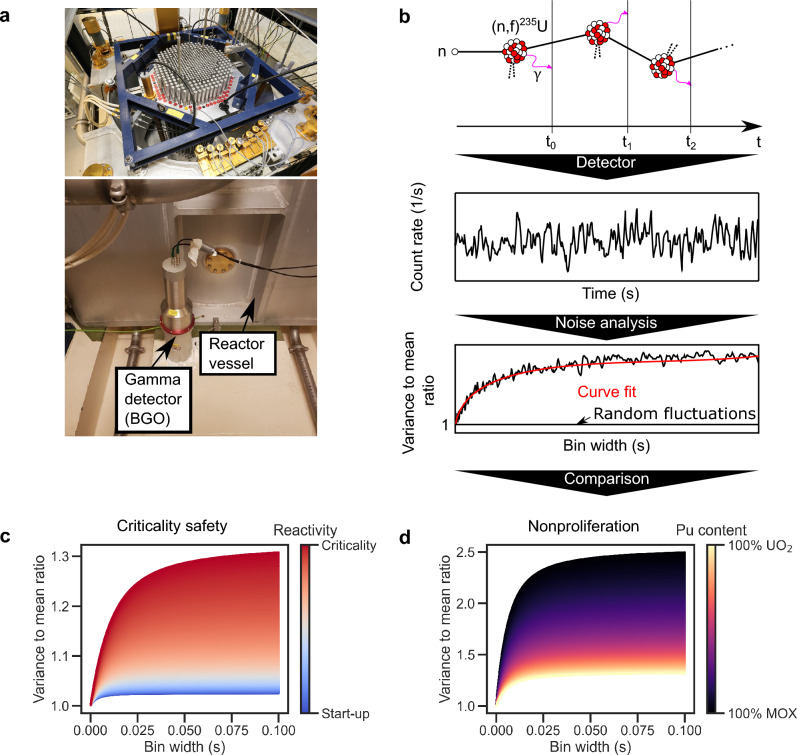
Concept of using correlations in fission chains to observe the criticality and/or fuel content of a nuclear reactor. (**a**) Above: Picture of the research reactor CROCUS. Below: Picture of a bismuth germanate scintillator next to the reactor vessel. (**b**) At each fission event in the reactor, prompt gamma rays are emitted that can be registered by a detector. Subsequent counts in the detector may stem from the same fission chain, leading to correlation in time. Analyzing the variance to mean ratio of the detector’s signal over varying intervals of time, one finds an excess variance compared to a purely random process. By fitting the variance to mean curve one can compare the result to previous records or code predictions for different reactor states in terms of criticality (**c**) or fuel composition (**d**). MOX refers to mixed-oxide fuel containing both uranium and plutonium (100% MOX meaning 8 wt% Pu). The figures were all created with simulated data.

In the interest of measuring both the current reactor state and changes in fuel composition, so called ’noise measurements’ may offer a method to obtain both. By exploiting the temporal correlation between subsequent detector’s counts one may directly observe a reactor’s fission chain propagation (see Fig. 1). A typical method in noise analysis is to measure the excess variance caused by the temporal correlation as compared to a strictly Poisson random process in which the variance is equal to the mean. Feynman et al. showed in 1956^11^ that the shape of this excess variance over an arbitrary bin size (Fig. 1b) can be expressed by an analytical function. A detector’s signal analyzed in this way can then be fitted with said expression to yield the so called ’prompt decay constant’ $\alpha$ of the system, a metric that captures the average prompt fission chain length. The result of this measurement may then be compared to previous data or code predictions of $\alpha$ to detect active reactor operation, reported schedule compliance, or potentially fuel composition changes.

The prompt fission chain length varies significantly, both with criticality as well as fuel composition of the system (Fig. 1c,d), and is thus informative for safety and safeguards applications. Noise measurements, however, face the challenge of requiring a minimum number of particles measured per fission occurring in their system^12,13^ and measurement times on the order of hours^14^. Hence, noise measurement applications are hitherto limited to in-core or in-vessel detection systems^15–18^, unable to fulfill safeguards needs due to the invasiveness.

Herein we present the application of high-efficiency gamma detectors to observe the criticality of a nuclear research reactor in ex-vessel locations using noise methods, well outperforming what is possible with neutron detectors. The system is based on bismuth germanate scintillators (BGO) weighing about 30 kg each, set at two distances outside the vessel of the research reactor CROCUS. Using the excess (co-)variance, we were able to measure the fission chain metric $\alpha$ accurately within minutes just outside the reactor vessel boundary. Furthermore, we were able to detect the excess variance at up to 7 meters distance, indicating that gamma noise methods may enable the use of noise techniques in reactor monitoring in a wide range of facilities, where in-core instrumentation is geometrically restricted or prohibited by regulation. Changes in reactor criticality affect $\alpha$ such that previous measurements or code predictions may be used for comparison and detection of deviations. Both the speed of and information from a noise measurement using gamma detectors therefore offer a cost-effective method for low-power reactor systems. Based on this proof of concept, we speculate that the ability to obtain criticality information at these distances may allow for noise methods to be adopted for safeguards applications, be it for fresh fuel or spent fuel verification, and reactor monitoring. This also includes potential novel applications, such as the monitoring of the state of damaged reactors or other instances with risks of re-criticality.

## Results

### Theory

Noise measurements typically rely on the so called ’point kinetics approximation’ to describe the time dependent behavior of the neutron flux *n*(*t*) in a fissile system^19,20^:

1 $$\begin{aligned} \frac{d n(t)}{dt} = \frac{\rho (t) -\beta _{\text {eff}}}{\Lambda } n(t) + \sum _i \lambda _i c_i, \end{aligned}$$

with the introduced quantities reactivity $\rho$, the emission energy weighted fraction of delayed neutrons $\beta _{\text {eff}}$, as well as the neutron generation time $\Lambda$. Equations for the delayed precursor concentrations $c_i$ and decay constants $\lambda _i$ can be derived from the associated Batman equation^20^.

The prompt decay constant $\alpha$ is the dominant eigenvalue of this system and is defined as:

2 $$\begin{aligned} \alpha = \frac{\beta _{\text {eff}}-\rho }{\Lambda } \end{aligned}$$

$\alpha$ therefore depends on the reactivity $\rho$, $\beta _{\text {eff}}$, and $\Lambda$.

The dependency of $\alpha$ on $\rho$ directly yields the use case for criticality safety and monitoring of a reactor (see Fig. 1c). With increasing reactivity the excess variance increases. A typical reference value of choice is the prompt decay constant at criticality ($\rho =0$), i.e. $\alpha = \frac{\beta _{\text {eff}}}{\Lambda }$.

The dependency of $\alpha$ at criticality on fuel composition hence stems from the changes in $\beta _{\text {eff}}$ and $\Lambda$. In the illustrated example (Fig. 1d) we used the values of the 100% UO_2_ compared to a 100% mixed oxide (MOX) core loading (about 8wt% of Pu) of the EOLE research reactor MISTRAL 1 and 2 programs^21^.

Noise methods are, by nature, non-invasive means (i.e. without reactor perturbation or dynamic experiments) to determine $\alpha$^22^. The nomenclature stems from the fact that a typical data set consists of detector signal time series, representing a stationary process. In analyzing the correlation in time induced by the fission chain (Fig. 1b) one may directly measure the prompt decay constant. A commonly used method is the Feynman-$\alpha$ excess variance analysis that formulates the fission chain correlations as excess variance over time compared to a strictly random process. The expression of the variance to mean (VTM) of detector counts *Z*(*t*) for a point reactor model is^11^:

3 $$\begin{aligned} \frac{\text {Var}(Z(t))}{\text {Mean}(Z(t))}= 1+ \frac{\varepsilon D_{\nu } }{(\rho -\beta _{\text {eff}})^2} \left( 1-\frac{1-e^{-\alpha t}}{\alpha t} \right) . \end{aligned}$$

with $\varepsilon$ the detector efficiency in counts per fission and $D_{\nu }=\overline{\nu _p(\nu _p-1)}/\overline{\nu _p}^2$ the Diven factor^23^. This formulation shows the aspect of modified variance due to branching: Instead of the variance being equal to the mean, as is the case for a Poisson process, we find an excess variance to mean above unity due to the fission chain reaction. This implies that at very short observation times the reactor noise signal appears Poisson-like (Var$(Z)=$Mean(*Z*)), whilst showing an increased variance for longer waiting times (Fig. 1b). Analogously, using two detectors the covariance to mean (CTM) expression reads (assuming equal efficiency $\epsilon _1=\epsilon _2=\epsilon$):

4 $$\begin{aligned} \frac{\text {Cov}(Z_{1,2}(t))}{\sqrt{\overline{Z_1(t) Z_2(t)}}}= \frac{\varepsilon D_{\nu } }{(\rho -\beta _{\text {eff}})^2} \left( 1-\frac{1-e^{-\alpha t}}{\alpha t} \right) . \end{aligned}$$

The CTM ratio offers some advantages to single detector methods, notably the filtering of uncorrelated (white) noise and thus a potentially higher signal-to-noise ratio^24,25^.

By applying a (co-)variance and mean calculation algorithm on detector data for variable bin widths (as outlined in the "Methods" section), one can then fit the VTM or CTM expression via a non-linear least squares approach to directly obtain the shape parameter $\alpha$. An important advance to previous experiments in this work is the use of gamma detectors instead of neutron detectors. The sufficient similarity of the noise information from gamma and neutron detectors in terms of measuring $\alpha$ has been shown experimentally in CROCUS with in-core detectors^17,18^. Theoretical considerations for VTM/CTM equations using gamma detectors were explored by Chernikova et al.^26^. This existing literature led to the hypothesis of gamma noise measurements being potentially superior to neutron noise measurements regarding precision and observable distances, alleviating the above-mentioned limitations of noise measurements.

**Figure 2:**
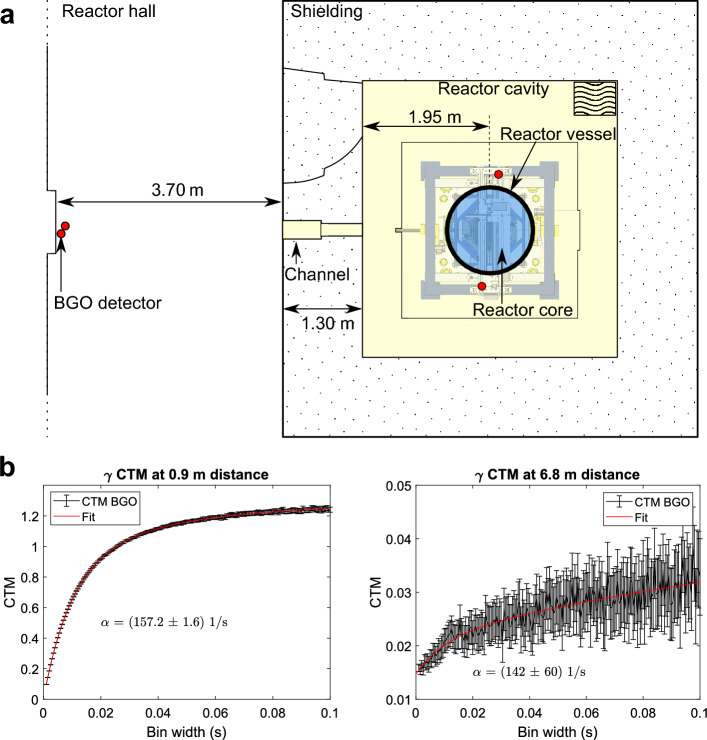
(**a**) Top view of the BGO detector locations for the gamma noise measurements. The red circles indicate BGO detector locations, whilst the blue disc demarcates the reactor vessel boundaries. (**b**) Resulting experimental covariance to mean (CTM) curves calculated from 2 hours of time series at each position fitted with the analytical expression yielding the prompt decay constant $\alpha$.

### Observation of the prompt decay constant outside the reactor vessel

The experiments were conducted in the CROCUS research reactor facility (see "Methods" section for details). The detector locations are shown in Fig. 2a. Two BGO scintillators were first set against the reactor vessel (Fig. 1a), and then at 6.8 m distance to the core center, which was the farthest location achievable within the CROCUS facility with a direct line of sight to the core center. To achieve this direct line of sight, the reactor’s irradiation channel in the west side of the concrete shielding was opened. The channel is centered at mid core height.

**Figure 3:**
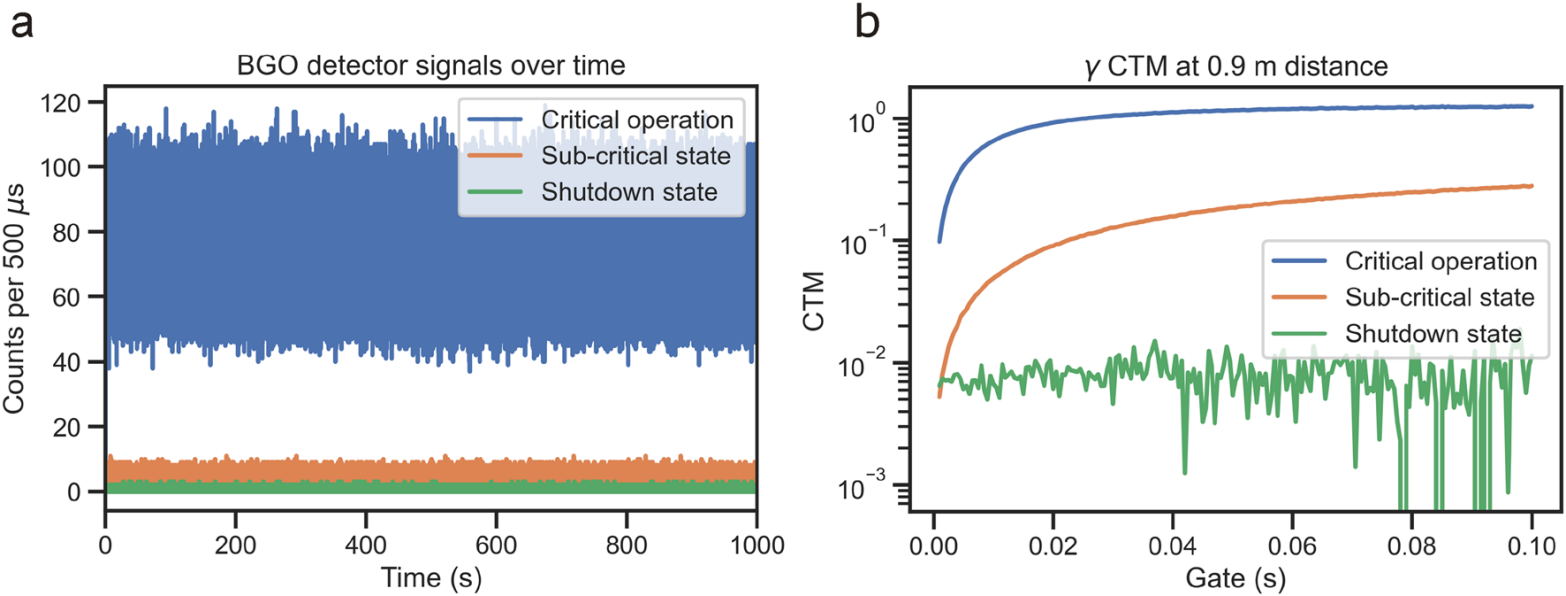
(**a**) Experimental count rates in a BGO detector at 0.9 m distance to the reactor for a critical operation at 20 mW, a sub-critical state during reactor startup (900 mm water level), and a shutdown state. (**b**) The corresponding CTM curves for each of the signals shown in (**a**).

In Fig. 2b we present the obtained CTM curves after two hours of measurement while the reactor was operated at criticality at around 50 mW power^27^. At the vessel boundary, after two hours of measurement time we find $\alpha =(157.2\pm 1.6)$ 1/s, i.e. with a relative error at around 1%. At the most distant location the prompt decay is found with a relative error around 40%, specifically $\alpha =(142\pm 60)$ 1/s, after 2 hours. To prove the general behavior of changing CTM with reactor state, we show in Fig. 3 the experimental count rates in a BGO detector over time and the corresponding CTM curves for a critical reactor, a sub-critical state during startup, and a shutdown reactor state.

Code prediction using the Serpent 2 Monte Carlo code^16,28^ and the ENDF/B-8.0^29^ nuclear data library yielded $\alpha =(157.3\pm 1.6)$ 1/s. At the ex-vessel location at 0.9 m we thus observe a relative difference to the mean value of less than 1%. At the far location at 6.9 m of $\sim$10% relative difference to the mean value of the code prediction.

**Figure 4:**
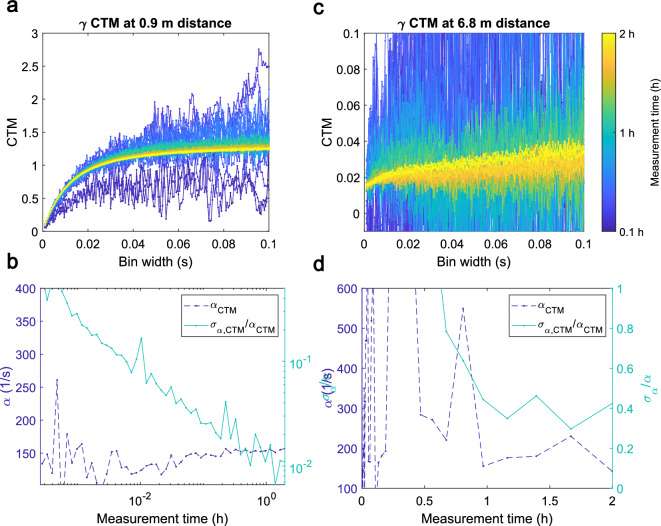
Overview of the convergence of the variance to mean curves as well the fit parameter $\alpha$ and its uncertainty $\sigma _{\alpha }$ over measurement time at 0.9 meters (**a**,**b**) and at 6.8 meters to the core (**c**,**d**).

In Fig. 4 we display the behavior of the CTM curves (a,c), and the fitted value for $\alpha$ and its uncertainty (b,d), over the measurement time. At 0.9 meters to the core we find that 10% parameter uncertainty is met after just 0.01 hours, or 72 seconds. 1% uncertainty is met after around 30 min. In the far location the uncertainties are much larger, well exceeding 100% until about 30 min of measurement time. Thereafter, the parameter mean and uncertainty converge towards the expected value.

## Discussion

Previous noise experiments in CROCUS using high-efficiency neutron detectors (1g of ^235^U fission chambers) showed that the correlations from fission chains are not statistically discernible beyond 20 cm distance to the fuel^13,25^. This was interpreted as an inherent limitation of neutron noise and may explain the little interest this method has garnered despite a long history. Experimental studies of gamma noise existed very early after the inception of noise analysis after 1960^30–32^, and we recently showed that in-core gamma noise can yield estimates of $\alpha$ with smaller uncertainty bounds than neutron noise experiments^17^. To our knowledge, no follow-up studies were conducted to quantify the spatial extent of gamma correlation information. Our results clearly show how gamma detectors outperform neutron detectors with respect to the distance at which the excess variance is meaningfully detectable, opening a new range of applicability for noise methods.

### Gamma noise for reactor monitoring

The noise method applied to reactor monitoring for safeguards and/or criticality monitoring may follow the following scheme: A gamma detector is set into the vicinity of a reactor. A prediction of $\alpha$ is provided either by code or a reference measurement. The detector signal can now be sampled in hourly batches and the variance to mean ratio per bin width calculated. The fitted $\alpha$ is then compared to the stored value. An arbitrarily defined deviation can then be used as a trigger for an inspection or closer monitoring of the facility.

Criticality monitoring using the system and method proposed is straightforward. Assuming a prompt decay constant at criticality of $\alpha =160$ 1/s, a sub-critical research reactor such as CROCUS (e.g. with control rods inserted, $\rho =-500\,pcm$) may be expected to exhibit $\alpha =256$ 1/s. A relative difference of 60% in $\alpha$ is clearly well detectable even at the farthest distance. A shutdown state is also clearly detectable, as shown in Fig. 3. Using the data for the close ex-vessel location, we may expect the accuracy of $\alpha$ to be sufficient after a measurement time of around one hour, with a general estimate of $\alpha$ with less than 10% uncertainty being available after just several minutes. We can nonetheless state that the location at 6.9 m may be inappropriate if information speed is to be of the order of hours.

For fuel composition changes the measurement is more complex. Supposing a full core fuel modification, which is unlikely to correspond to a clandestine fuel deviation scenario, a significant difference in $\alpha$ or the VTM/CTM plateau is expected. Taking the differences in predicted $\alpha$ for the case example of a UO_2_ and MOX core as used in Fig. 1d, namely $\alpha =288$ 1/s for UO_2_ fuel and $\alpha =276$ 1/s for MOX with 8wt% of Pu^21^, we find that the observation of fuel changes will require the detection of a relative change in $\alpha$ of 4% or less. The more sensitive parameter with fuel composition is arguably the delayed neutron fraction $\beta _{\text {eff}}$: For a UO_2_ it is around 0.0075, whilst for a full MOX core it is 0.0035. This relative difference of more than 50% is the main visual difference in Fig. 1d, as $\beta _{\text {eff}}$ also affects the amplitude of the excess variance. The amplitude term of gamma noise VTM/CTM curves is however a more complex expression that includes gamma multiplicities, delayed gamma rays, and the gamma ray absorption^26^. Future work would therefore be needed to investigate whether the gamma-ray induced terms are significant, and to quantify whether a relevant signal can be detectable for a given fuel composition change scenario.

### Comparison to other systems

Past work used neutron detectors for neutron flux measurements at up to 100 m to a 135 MW reactor^7^ and stated that the fuel composition can be tracked with this method. As the detectors only measure flux information, it is reasonable to assume that the readout can be manipulated by intentionally placing a neutron source to perturb the readout.

Noise methods are in principle resistant to such tampering, as such a tampered signal would show a variance to mean ratio of one (i.e. a Poisson distribution of events), and not the excess variance due to the fission chain. The difficulty of mimicking the exact correlations that a reactor produces emphasizes the advantage of using noise methods. Similar considerations are true for power distribution imaging^33^ beyond the reactor shielding, that also lack explicit information on the criticality of the system.

Antineutrino-based systems are potentially the most tamper-proof, as antineutrino fluxes cannot be feasibly shielded nor falsified by other sources. Autonomous reactor monitoring using antineutrinos requires the estimation of the incoming neutrino spectrum in order to infer fuel composition changes^8,34,35^. Measurements of antineutrino spectra are still under active research^9^, and the large associated uncertainties and low count rates lead to an effective speed of information of the order of weeks at best. Another challenge for antineutrino detectors is the required detector volume to reach the desired levels of signal. For instance, the PROSPECT antineutrino detector has 3000 l active volume of scintillator, requiring hundreds of photomultiplier tubes (PMTs) coupled to it^36^. The thus associated challenges of construction, mobility, and maintenance of sensitive parts of the detector put the widespread practical use into question. An initial cost of about $5 million for such a system^37^ is a major limitation as well. Furthermore, the background due to solar neutrinos and signal corrections due to neutrino oscillations are to be carefully considered and add another layer of complexity. These systems seem nonetheless the best technology for large power reactors with $$\sim $$GW of power.

Noise methods using gamma rays once more may offer a cheaper (the setup used here cost less than 80 k$ total), more compact, less complex system that provides direct information on the fission chain propagation. To perform noise measurements the detectors need not be calibrated beyond simple visual inspection of oscilloscope signals, as the information is coded in the timing between pulses, offering a comparatively fast setup. The proposed gamma noise system requires two BGO scintillator crystals at 30 kg each and two PMTs (see "Methods" section), leading to timing information reaching relevant precision within minutes or hours, depending on the distance to the reactor.

A key limitation to applying fission chain correlation analysis to reactor observation is the tendency of thermal-hydraulics noise (i.e. mechanical vibrations of the system due to coolant flow) to overpower the observed fission correlation signals^38,39^. The herein proposed technique is therefore likely to not provide useful information in reactors operating at>100 kW, yet this remains to be tested.

A common application of correlation measurements for nuclear safeguards is neutron multiplicity counting^40,41^, usually used for the accountancy of small quantities of Pu. Gamma-ray multiplicity counting has been theoretically and experimentally explored^42^, yet a conclusive theory or practical apparatus with similar performance to neutron counting systems is yet to emerge. Commonly identified problems, such as radiative capture gamma rays or self-absorption of gamma rays in the high Z sample, do not appear to significantly affect the measurements presented herein. A dedicated research project investigating correlations arising from different sources using Monte Carlo codes appears promising.

### Safeguarding future nuclear systems

A recent technological trend in nuclear power installations is the shift towards small modular reactors (SMRs), systems with less than 300 MW_e_ power according to the IAEA definition^43^. They promise a less capital intensive and more scalable power generation, with designs in advanced stages of construction found in Argentina, China, and Russia. SMRs however fall into the same category as research reactors, and as such are harder to monitor using standard safeguard techniques^6^. This is highlighted for instance in the French nuclear program, which likely relied on <MW reactors (zero power reactors such as the Zoé^44^) for plutonium production^45,46^.

SMRs are more challenging to monitor for fuel changes than larger systems, and this directly impacts the usefulness of the autonomous systems based on neutrons or antineutrinos. As we have demonstrated, the distance at which explicit information of the fission chains can be detected with gamma rays extends to the order of meters outside a research reactor vessel. Smaller reactor systems are also more likely to not be dominated by thermo-hydraulic noise sources (especially during startup), and are more likely to follow the mentioned point-like behavior. The measurement time could hereby be as small as minutes to hours, minimizing the window of opportunity for misuse.

Future research should aim at replicating our results in other and more challenging reactor geometries to illuminate the limits and lend evidence of the applicability. The noise method may be extended to investigate the observability of gamma noise in other relevant domains of criticality safety, such as spent fuel pool monitoring. Here the fuel could be interrogated with a neutron source and the resulting excess variance could indicate if fuel has been removed. To date, the gamma correlation aspect remains poorly understood. This could equivalently be extended to accident scenarios, where the exact state of a reactor may be unknown. For instance, in the Units 1-3 in the Fukushima Daiichi power plant the exact core damage is still to be quantified^47,48^. Current efforts include cosmic muon radiography^49^, which gave first insights into the reactor internal status. The analysis of the time correlations in gamma signals may reveal in what state the core remains are.

## Methods

### The CROCUS zero power research reactor

**Figure 5:**
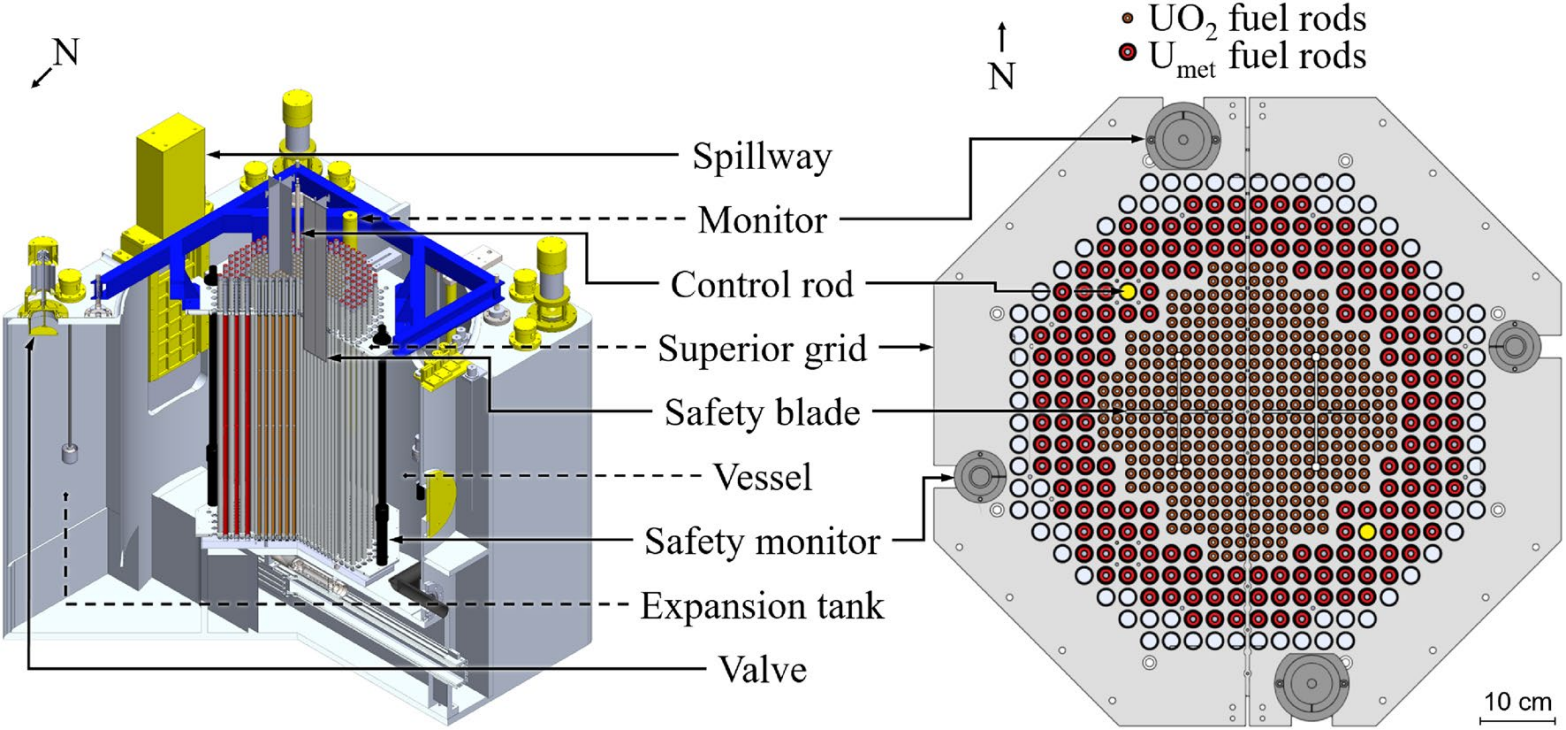
Schematic isometric view of the CROCUS reactor (left), and top view of the core configuration (right).

A full description of the core can be found in the International Reactor Physics Experiments Handbook (IRPhE)^50^. CROCUS is a two-zone, uranium-fueled, H_2_O-moderated critical assembly operated by the Laboratory for Reactor Physics and Systems Behaviour (LRS) at the Swiss Federal Institute of Technology Lausanne (EPFL). With a maximum power of 100 W it is considered a zero power reactor. The core is approximately cylindrical in shape with a diameter of about 58 cm and a height of 100 cm. Two different kinds of fuel rods make up the reactor core of CROCUS (see Fig. 5). The central zone is loaded with 336 UO_2_ fuel rods (1.806 wt.%-enriched), set in a square lattice with a pitch of 1.837 cm. The peripheral zone is loaded with up to 176 thicker, U_met_ fuel rods (0.947 wt.%-enriched) with a pitch of 2.917 cm, also in a square lattice. All fuel rods are cladded with aluminum and are maintained in a vertical position by the upper grid and lower grid plates spaced 100 cm apart. The active fuel length starts at the top surface of the lower cadmium layer and extends to 100 cm. The core is in an aluminum water tank, its diameter is 130 cm and thickness is 1.2 cm. Demineralized light water is used as moderator and reflector. Reactivity is nominally controlled by a variation of the water level using a spillway with an accuracy of 0.1 mm (equivalent to 0.4 pcm) and optionally by means of two control rods containing naturally enriched boron carbide (B_4_C) sintered pellets located in diagonal symmetry within the outer fuel zone.

### Gamma noise experiments

In this work we used two large BGO detectors of the LEAF system in CROCUS. For more details and their calibration the reader is referred to^51^. With their size (cylindrical crystal, diameter of 127 mm and 254 mm height) and scintillator typing (high Z) they are designed to count most photons (>95%) below 10 MeV that enter the crystal volume. Both detectors house a Photonis 5” Type XP4578 PMT. The post-PMT signal amplification and high voltage (HV) supply to the PMT is handled by a Mirion DSA-LX^52^. The connection between the PMT and the DSA-LX amplification unit for HV and signal was established with 30 m long BNC-HV and BNC cables that were also used during calibration. For consistent and reproducible signals, we chose the settings for the amplification electronics (DSA-LX) for all experiments to be as follows:

- Rise time of 0.2 $\mu$s, with a 0.0 $\mu$s flat top setting.
– 1260 V of HV to the PMTs.
- Lower level discrimination at 0.5% of the maximum channel (2^14^).
- Coarse gain of 6.4 for both detectors.

The respective gamma ray spectra of the detectors and their lower level threshold optimization are presented in other work^17,25,51,53^. The detector counts were registered using a multi-channel buffer card on an in-house developed PC^25,54^. The signals were acquired with a sampling rate of 2000 Hz, i.e. an acquisition bin width of 0.5 ms.

### Variance to mean estimation and fitting algorithms

VTM and CTM curves were estimated using a custom algorithm written in MATLAB2019b^55^ up to a maximum bin width of 0.1 s. For each given bin width (which needs to be an integer multiple of the acquisition bin width of 0.5 ms) the signal was summed into the new bins of width *t*. The variance and mean of the resulting vector is then the VTM/CTM at bin width *t*.

The VTM/CTM curves were fitted with the response function (Eq. 4) using a non-linear least squares approach using a trust region optimizer, with inverse observed variance as weight. The explicit form of the fitted function was

5 $$\begin{aligned} \frac{\text {Cov}(Z_{1,2}(t))}{\sqrt{\overline{Z_1(t)Z_2(t)}}} = Y(t) = a \left( 1-\frac{1-e^{-\alpha t}}{\alpha t} \right) + c, \end{aligned}$$

with *a* fitting for the amplitude term and *c* accounting for dead time effects^56^. A linear term in the calculated (co-)variance to mean curves was found to be caused by signal spikes in the time series of detector 1 that were attributed to electronic noise. These spikes were removed manually.

The uncertainties in each point of *Y* were calculated via^54^:

6 $$\begin{aligned} \sigma _Y(t) = \frac{(Y+1)}{\sqrt{N}} \cdot \sqrt{\frac{Y+1}{\sqrt{\overline{Z_1(t)Z_2(t)}}}+\frac{2N}{N-1}}, \end{aligned}$$

with *N* the number of acquisition bins widths amounting to the total bin width *t*.

### Applying neutron noise equations to gamma noise experiments

The theoretical reasoning as to why gamma noise follows approximately the neutron induced correlations has been explored by other authors^26^. They found that the VTM and CTM expressions for a gamma detector signal will take the following shape:

7 $$\begin{aligned} \frac{\text {Cov}(Z_{1,2}(t))}{\sqrt{\overline{Z_1(t)Z_2(t)}}} = Y(t) = a \left( 1-\frac{1-e^{-\alpha t}}{\alpha t} \right) + b \left( 1-\frac{1-e^{-\alpha _{\gamma } t}}{\alpha _{\gamma } t} \right) + c, \end{aligned}$$

We find two terms: one is governed by the excess variance due to the fission chain propagation and thus has the shape factor $\alpha$. The second term describes the gamma ray propagation which is characterized by a ’prompt gamma ray decay constant’, $\alpha _{\gamma }$. Consequently, the form of an observed VTM/CTM function from a gamma detector is influenced by both $\alpha$ and $\alpha _{\gamma }$. A core assumption in applying the analytical VTM/CTM expression for gamma noise is $\alpha \lll \alpha _{\gamma }$, leading to a negligible contribution by the gamma ray propagation to the observed correlation, and thus the absorption of the gamma term into the constant *c*.

The prompt chain correlations in a system such as CROCUS decay to the delayed and uncorrelated baseline after around 20 ms. Gamma propagation in CROCUS, even to a detector at 7 meters distance, happens within 24 ns. Any gamma propagation that would result in a visible correlation must occur within such a time window, corresponding to a rough lower limit of

8 $$\begin{aligned} \alpha _{\gamma }> \frac{1}{ 2\pi \cdot 24{\textbf { ns}}} \approx 6.6\cdot 10^6 \frac{1}{\text {s}}. \end{aligned}$$

This hypothesis is expected to be valid for critical moderated systems and moderated systems approaching criticality. Further theoretical work is needed to quantify the magnitude of the gamma propagation term. This is in the interest of knowing the impact of fuel composition changes on the observed VTM/CTM plateau value, but also for noise analysis in fast assemblies such as Godiva^57^ or the BeRP ball Pu sphere^58^, where prompt chains decay within hundreds of nanoseconds.

## Data Availability

The data generated and/or analyzed in this study are available from the corresponding author upon reasonable request.

## Code availability

The code used to process the data is available from the corresponding author upon reasonable request.

## References

[1] Pörtner, H.-O. et al. IPCC: Climate Change 2022: Impacts, Adaptation, and Vulnerability. Contribution of Working Group II to the Sixth Assessment Report of the Intergovernmental Panel on Climate Change (Cambridge University Press, 2022).

[2] Ming A (2021). Key messages from the IPCC AR6 climate science report. Cambridge Open Engage.

[3] Kober T, Schiffer H-W, Densing M, Panos E (2020). Global energy perspectives to 2060 - WEC’s world energy scenarios 2019. Energy Strat. Rev..

[4] Anzelon, G. Antineutrino reactor monitoring in the context of IAEA safeguards. Workshop on Applied Antineutrino Physics (AAP 2018).

[5] IAEA Department of Safeguards (2013). Long-term R &D plan, 2012–2023, STR-375.

[6] Whitlock J, Sprinkle J (2014). Proliferation resistance considerations for remote small modular reactors. Nucl. Rev..

[7] van der Ende BM, Li L, Godin D, Sur B (2019). Stand-off nuclear reactor monitoring with neutron detectors for safeguards and non-proliferation applications. Nat. Commun..

[8] Stewart C, Abou-Jaoude A, Erickson A (2019). Employing antineutrino detectors to safeguard future nuclear reactors from diversions. Nat. Commun..

[9] Almazán H (2021). First antineutrino energy spectrum from 235U fissions with the STEREO detector at ILL. J. Phys. G Nucl. Part. Phys..

[10] Ashenfelter J (2019). Measurement of the antineutrino spectrum from U235 fission at HFIR with PROSPECT. Physical Review Letters.

[11] Feynman RP, De Hoffmann F, Serber R (1956). Dispersion of the neutron emission in U-235 fission. J. Nucl. Energy.

[12] Pázsit, I. & Demazière, C. Noise Techniques in Nuclear Systems (Chapter in Handbook of Nuclear Engineering-Reactors of Generation II, 2010).

[13] Pakari O (2018). Investigation of Spatial Effects on Neutron Noise Measurements in the Zero Power Reactor CROCUS.

[14] Geslot, B. et al. Pile noise experiment in MINERVE reactor to estimate kinetic parameters using various data processing methods. In Advancements in Nuclear Instrumentation Measurement Methods and their Applications (ANIMMA), 2015 4th International Conference on, 1–8 (IEEE, 2015).

[15] Gilad E (2015). Experimental estimation of the delayed neutron fraction $$\beta _{\text{eff}}$$ of the MAESTRO core in the MINERVE zero power reactor. J. Nucl. Sci. Technol..

[16] Pakari O, Lamirand V, Perret G, Frajtag P, Pautz A (2018). Kinetic parameter measurements in the crocus reactor using current mode instrumentation. IEEE Trans. Nucl. Sci..

[17] Pakari OV, Lamirand V, Mager T, Frajtag P, Pautz A (2022). High accuracy measurement of the prompt neutron decay constant in CROCUS using gamma noise and bootstrapped uncertainties. Ann. Nucl. Energy.

[18] Darby FB (2023). Neutron-gamma noise measurements in a zero-power reactor using organic scintillators. IEEE Trans. Nucl. Sci..

[19] Williams MMR (2013). Random Processes in Nuclear Reactors.

[20] Ott KO, Neuhold RJ (1985). Introductory Nuclear Reactor Dynamics.

[21] Yamamoto T, Iwahashi D, Sakai T (2020). Applying the continuous-energy Monte Carlo calculation code, MVP3, to analysis of kinetic parameters measured for light-water moderated UO2 and MOX cores of the TCA and EOLE critical facilities. Nucl. Sci. Technol..

[22] Uhrig RE (1970). Random Noise Techniques in Nuclear Reactor Systems.

[23] Diven B, Martin H, Taschek R, Terrell J (1956). Multiplicities of fission neutrons. Phys. Rev..

[24] Pázsit I, Pál L (2007). Neutron Fluctuations: A Treatise on the Physics of Branching Processes.

[25] Pakari, O. V. Experimental and numerical study of stochastic branching noise in nuclear reactors. Ph.D. thesis, École polytechnique fédérale de Lausanne (EPFL) (2020). 10.5075/EPFL-THESIS-8336.

[26] Chernikova D (2015). The neutron-gamma Feynman variance to mean approach: Gamma detection and total neutron-gamma detection (theory and practice). Nucl. Instrum. Methods Phys. Res. Sect. A Accel. Spectrom. Detect. Assoc. Equip..

[27] Lamirand V, Laureau A, Pakari O, Frajtag P, Pautz A (2020). Power calibration methodology at the CROCUS reactor. EPJ Web Conf..

[28] Leppänen J, Aufiero M, Fridman E, Rachamin R, van der Marck S (2014). Calculation of effective point kinetics parameters in the Serpent 2 Monte Carlo code. Ann. Nucl. Energy.

[29] Brown D (2018). ENDF/b-VIII.0: The 8th major release of the nuclear reaction data library with CIELO-project cross sections, new standards and thermal scattering data. Nucl. Data Sheets.

[30] Lehto WK, Carpenter JM (1967). Reactor noise measurements using gamma rays. Nucl. Sci. Eng..

[31] Kenney ES, Schultz MA (1969). Local in-core power measurements with out-of-core gamma detectors. Nucl. Appl..

[32] Bärs B, Markkanen E (1972). Reactor parameters from neutron and gamma-ray noise measurements. Nucl. Sci. Eng..

[33] Beaumont JS, Mellor MP, Villa M, Joyce MJ (2015). High-intensity power-resolved radiation imaging of an operational nuclear reactor. Nat. Commun..

[34] Mueller TA (2011). Improved predictions of reactor antineutrino spectra. Phys. Rev. C.

[35] Boireau G (2016). Online monitoring of the OSIRIS reactor with the NUCIFER neutrino detector. Phys. Rev. D.

[36] Ashenfelter J (2016). The PROSPECT physics program. J. Phys. G Nuclear Part. Phys..

[37] Wright K (2020). Neutrino detectors for national security. APS Phys..

[38] Cacuci DG (2010). Handbook of Nuclear Engineering.

[39] Spriggs GD (1994). The reactor noise threshold. Nucl. Sci. Eng..

[40] Di Fulvio A (2017). Passive assay of plutonium metal plates using a fast-neutron multiplicity counter. Nucl. Instrum. Methods Phys. Res. Sect. A Accel. Spectrom. Detect. Assoc. Equip..

[41] Darby FB, Hua MY, Pakari OV, Clarke SD, Pozzi SA (2023). Multiplicity counting using organic scintillators to distinguish neutron sources: An advanced teaching laboratory. Am. J. Phys..

[42] Enqvist A, Flaska M, Dolan JL, Chichester DL, Pozzi SA (2011). A combined neutron and gamma-ray multiplicity counter based on liquid scintillation detectors. Nucl. Instrum. Methods Phys. Res. Sect. A Accel. Spectrom. Detect. Assoc. Equip..

[43] Subki H (2020). Advances in Small Modular Reactor Technology Developments.

[44] Couturier J, Hassan Y, Grolleau E, Couturier J, Hassan Y, Grolleau E (2021). Evolution of the French research reactor "fleet". Element of Nuclear Safety.

[45] Kemp RS (2019). The Iran nuclear deal as a case study in limiting the proliferation potential of nuclear power. Nat. Energy.

[46] Hecht G, McKenzie F (2000). The radiance of France: Nuclear power & national identity after World War II. Can. J. Hist..

[47] Tokyo Electric Power Co, Inc, Tokyo (Japan). The evaluation status of reactor core damage at Fukushima Daiichi NPS units 1 to 3. Handouts at press conference on 2011-11-30 (2011).

[48] Ono A (2021). Fukushima Daiichi decontamination and decommissioning: current status and challenges. Ann. ICRP.

[49] Fujii, H. et al. Investigation of the unit-1 nuclear reactor of Fukushima Daiichi by cosmic muon radiography. Progress of Theoretical and Experimental Physics **2020**, 10.1093/ptep/ptaa027 (2020).

[50] Kasemeyer, U., Früh, R., Paratte, J. M. & Chawla, R. Benchmark on kinetic parameters in the CROCUS reactor. International Reactor Physics Experiments Handbook (IRPhE), (2007).

[51] Pakari O (2021). First in-core gamma spectroscopy experiments in a zero power reactor. EPJ Web Conf..

[52] DSA-LX, Digital signal analyzer. https://www.mirion.com/products/dsa-lx-digital-signal-analyzer.

[53] Pakari OV (2024). Gamma-ray spectroscopy in low-power nuclear research reactors. J. Nucl. Eng..

[54] Perret, G. Tm-41-14-02 rev. 1: Decay constant and delayed neutron fraction measurements in CROCUS. Tech. Rep., ERP, LRS, Paul Scherrer Institut (2014).

[55] MATLAB. 9.7.0.1190202 (R2019b) (The MathWorks Inc., 2019).

[56] Hazama T (2003). Practical correction of dead time effect in variance-to-mean ratio measurement. Ann. Nucl. Energy.

[57] Goda J (2021). A New Era of Nuclear Criticality Experiments: The First 10 Years of Godiva IV Operations at NCERC. Nucl. Sci. Eng..

[58] Hua MY (2020). Rossi-alpha measurements of fast plutonium metal assemblies using organic scintillators. Nucl. Instrum. Methods Phys. Res. Sect. A Accel. Spectrom. Detect. Assoc. Equip..

